# High-throughput functional analysis provides novel insight into type VII secretion in *Staphylococcus aureus*

**DOI:** 10.1098/rsob.240060

**Published:** 2024-08-14

**Authors:** Yaping Yang, Aaron A. Scott, Holger Kneuper, Felicity Alcock, Tracy Palmer

**Affiliations:** ^1^ Newcastle University Biosciences Institute, Newcastle University, Newcastle upon Tyne NE2 4HH, UK

**Keywords:** type VII secretion system, *Staphylococcus aureus*, bioluminescence, NanoBit, toxin, accessory factors

## Abstract

Successful colonization by the opportunistic pathogen *Staphylococcus aureus* depends on its ability to interact with other microorganisms. *Staphylococcus aureus* strains harbour a T7b subtype of type VII secretion system (T7SSb), a protein secretion system found in a wide variety of Bacillota, which functions in bacterial antagonism and virulence. Assessment of T7SSb activity in *S. aureus* has been hampered by low secretion activity under laboratory conditions and the lack of a sensitive assay to measure secretion. Here, we have utilized NanoLuc binary technology to develop a simple assay to monitor protein secretion via detection of bioluminescence. Fusion of the 11 amino acid NanoLuc fragment to the conserved substrate EsxA permits its extracellular detection upon supplementation with the large NanoLuc fragment and luciferase substrate. Following miniaturization of the assay to 384-well format, we use high-throughput analysis to demonstrate that T7SSb-dependent protein secretion differs across strains and growth temperature. We further show that the same assay can be used to monitor secretion of the surface-associated toxin substrate TspA. Using this approach, we identify three conserved accessory proteins required to mediate TspA secretion. Co-purification experiments confirm that all three proteins form a complex with TspA.

## 1. Background

The opportunistic pathogen *Staphylococcus aureus* is a major cause of both nosocomial and community-acquired infections. It is a frequent colonizer of animals and humans, and infections can result in a range of life-threatening diseases such as pneumonia, endocarditis and sepsis. It is notorious for developing resistance to frontline anti-microbials, with methicillin resistance now widespread and vancomycin resistance also increasing [1,2]. Like many Gram-positive bacteria, *S. aureus* carries a specialized protein secretion machinery called the type VII secretion system (T7SS), which it utilizes to secrete anti-bacterial toxins targeting competitor bacteria (reviewed in [3]). T7SS-mediated antagonism is likely to be important for colonization [4], and the *S. aureus* T7SS has been shown to be required for virulence in mouse infection models [5–9]. There are several distinct T7SS subtypes, of which the T7SSa and T7SSb are the best characterized [10]. The T7SSb is widely distributed among Bacillota and has been studied to date in the species of *Streptococcus*, *Staphylococcus*, *Bacillus* and *Enterococcus* [8,11–13]. However, in many cases, functional and mechanistic studies have been hindered by low secretion activity in laboratory conditions.

The *S. aureus* T7SSb is encoded on the core genome at the *ess* locus, and four variant locus types have been identified, termed *essC1* to *essC4*. The genes at the 5′ end of the *ess* locus are highly conserved and encode core components of the secretion machinery, while the 3′ end codes for different complements of variant-specific substrate and accessory genes [4,14]. The primary component of the T7SSb transmembrane channel is the ATPase EssC, inferred from its homology to the EccC component of the mycobacterial T7SSa channel whose structure has been solved by cryo-electron microscopy [15,16]. EssC has a conserved N-terminus, two transmembrane domains which likely form the secretion channel and four C-terminal ATPase domains, the latter two of which are variant-specific and provide substrate specificity [17]. Three additional membrane proteins, EsaA, EssA and EssB, and two small globular proteins, EsxA and EsaB, are further essential components of the T7SSb secretion system [6,8,18].

In addition to being a core component of the T7SSb secretion system, EsxA is itself a T7SS secretion substrate. EsxA secretion is essential for the export of other T7SSb substrates, and it appears to be co-secreted with at least some of them [8,19]. EsxA is a member of the WXG100 protein family, which are helical hairpin proteins that often, but not always, harbour a central W-x-G sequence motif [20,21]. In addition to EsxA, other WXG100-like proteins, with more specialized functions, are also found in T7SSb^+^ strains. These are usually encoded at genetic loci with genes for larger T7SSb-secreted toxins. T7SSb toxins contain an LXG domain, which can be located at either the protein N-terminus (e.g. TelB, EsaD and TspA) or C-terminus (e.g. TslA) [6,11,18,22–25]. Prior to secretion, the co-encoded WXG100-like proteins, which have been termed Laps (for LXG-associated α-helical proteins), bind to the LXG domain of the cognate toxin partner. This interaction generates a secretion-competent rod-shaped complex, and conserved sequence motifs on both the LXG domain and one of the Laps constitute a T7SSb-targeting signal [23,24,26,27]. In some instances, a globular protein is also encoded at the toxin genetic locus, which also facilitates toxin secretion [24,27].

TspA is a membrane-depolarizing toxin with anti-bacterial activity that is encoded by all *S. aureus* strains analysed to date. Following secretion, TspA associates with the cell surface and can only be released experimentally by digestion of the cell wall [22]. Unlike other characterized LXG toxins, no WXG100-like partner proteins are encoded at the *tspA* locus, and its secretion requirements are unclear. Assessing secretion by the *S. aureus* T7SS is hampered by low secretion activity in laboratory growth media. Moreover, the lack of cleavable signal peptides on T7SSb substrates means that there is no size difference between the exported and cytosolic forms making cellular lysis a confounding issue, particularly where cell wall digestion is also required. To circumvent these difficulties, we have developed a novel secretion assay that makes the use of NanoLuc binary technology (NanoBit) [28]. We show that this is a robust reporter system that can be used to monitor both secreted and cell surface-localized T7SSb substrates, which is amenable to high-throughput analysis. Using this assay, we identify three genes that are required for efficient export of TspA and demonstrate that the encoded proteins directly interact with the toxin. We predict that this assay will be of considerable utility in future studies of the T7SSb.

## 2. Methods

### 2.1. Bacterial strains, plasmids and growth conditions

*Escherichia coli* was cultured in LB medium (Melford), and *S. aureus* was cultured in tryptic soy broth (TSB; Oxoid) with chloramphenicol (10 μg ml^−1^) where required. *Escherichia coli* strains DH5α [*Δ(argF-lac)169, φ80dlacZ58(M15), ΔphoA8, glnX44(AS), deoR481, rfbC1, gyrA96(NalR), recA1, endA1, thiE1 and* hsdR17] and JM110 *rpsL thr leu thi lacY galK galT ara tonA tsx dam dcm glnV44 Δ(lac-proAB*) e14- [F’ *traD36 proAB^+^ lacI^q^ lacZ*ΔM15] *hsdR17*(rK^−^mK*^+^*) were used for cloning and preparation of plasmids for electroporation, respectively, and M15 harbouring pREP4 (*F-, lac, ara, gal, mtl* [*KanR, lacI*]) was used for protein overproduction. *Staphylococcus aureus* strains and plasmids used are listed in tables 1 and 2 respectively. Chromosomal deletion of *essC* was accomplished by allelic exchange using plasmid pIMAY [37] carrying the *essC* flanking regions. Insertion of the pep86 tag on the chromosome of COL and COLΔ*essC* used an updated system with plasmid pIMAY-Z [38]. pIMAY plasmids were created using standard restriction cloning techniques, and pIMAY-Z-esxApep86 was created using HiFi assembly (NEB). Plasmids pRab11-esxApep86 and pRab11-tspA_1-328_-pep86_2603_2602_2601 for NanoLuc secretion assays, and plasmid pQE70-_Tstrep_SACOL2603_myc_-2602-2601_HA_-_his_TspA for protein purification were created using HiFi assembly (NEB, E0554S). *SACOL2603*, *SACOL2602* and *SACOL2601* were individually deleted from pRab11-tspA_1-328_-pep86_2603_2602_2601 using the Q5 site-directed mutagenesis kit (NEB). Oligonucleotides are listed in electronic supplementary material, table S1.

**Table 1.** *Staphylococcus aureus* strains used in this study.

strain	description	reference
COL	MRSA, *agr, essC1* variant strain	[29,30]
COLΔ*essC*	COL with markerless deletion of *essC*	[22]
RN6390	NCTC8325 derivative. *rbsU, tcaR,* cured of φ11, φ12, φ13. *essC1* variant strain	[31,32]
RN6390Δ*essC*	RN6390 with markerless deletion of *essC*	[8]
RN6390*ΔesxA*	RN6390 with markerless deletion of *esxA*	[8]
JP5347	human isolate, *essC1* variant strain	this work
JP5347Δ*essC*	JP5347 with markerless deletion of *essC*	this work
MRSA252	nosocomial HA-MRSA isolate, representative of epidemic MRSA-16. *essC2* variant strain	[33]
MRSA252Δ*essC*	MRSA252 with markerless deletion of *essC*	this work
10.1252 .X	livestock-associated ST398 isolate. *essC3* variant strain	[34]
10.1252.XΔ*essC*	10.1252 .X with markerless deletion of *essC*	this work
EMRSA15	EMRSA-15 clonal complex 22 human isolate. *essC4* variant strain.	[35]
EMRSA15Δ*essC*	EMRSA15 with markerless deletion of *essC*	this work
COL*_esxA-pep86_*	Pep86 coding sequence (VSGWRLFKKIS) fused to the 3′ end of *esxA* in COL	this work
COLΔ*essC_esxA-pep86_*	Pep86 coding sequence (VSGWRLFKKIS) fused to the 3′ end of *esxA* in COLΔ*essC*	this work

**Table 2.** Plasmids used in this study.

plasmid	details	ref./source
pRab11	*E. coli*/*S. aureus* shuttle vector, carries P_xyl/tet_ for inducible expression. Amp^r^, cml^r^	[36]
pRab11-esxApep86	for ATC inducible expression of EsxA with a C-terminal pep86 tag (VSGWRLFKKIS)	this work
pRab11-pep86trxA	for ATC inducible expression of TrxA with a N-terminal pep86 tag (MVSGWRLFKKIS)	this work
pRab11-CBED-DG	plasmid for overproduction of the *essC1* substrate EsaD with a C-terminal HA tag, along with its immunity protein EsaG and secretion factors EsxB, EsxC, EsxD and EsaE	[24]
pRab11-tspA_1-328_-pep86_2603_2602_2601	for ATC inducible production of TspA_NT_ with a C-terminal pep86 tag (VSGWRLFKKIS), along with LapT1 (SACOL2603), LapT2 (SACOL2602) and SACOL2601	this work
pRab11-tspA_1-328_-pep86_2602–2601	for ATC inducible production of TspA_NT_ with a C-terminal pep86 tag (VSGWRLFKKIS), along with LapT2 (SACOL2602) and SACOL2601	this work
pRab11-tspA_1-328_-pep86_2603_2601	for ATC inducible production of TspA_NT_ with a C-terminal pep86 tag (VSGWRLFKKIS), along with LapT1 (SACOL2603) and SACOL2601	this work
pRab11-tspA_1-328_-pep86_2603–2602	for ATC inducible production of TspA_NT_ with a C-terminal pep86 tag (VSGWRLFKKIS), along with LapT1 (SACOL2603) and LapT2 (SACOL2602)	this work
pIMAY	*E. coli*/*S. aureus* shuttle vector, temperature sensitive, cml^r^	[37]
pIMAYessC	pIMAY carrying the flanking regions of *essC1*	[8]
pIMAYessC-MRSA252	pIMAY carrying the flanking regions of *essC2*	this work
pIMAYessC-ST398	pIMAY carrying the flanking regions of *essC3*	this work
pIMAYessC-EMRSA15	pIMAY carrying the flanking regions of *essC4*	this work
pIMAY-Z	*E. coli*/*S. aureus* shuttle vector, temperature sensitive, cml^r^	[38]
pIMAY-Z_esxApep86	pIMAY-Z carrying *esxA-pep86* and chromosomal flanking regions	this work
pBAD−_6H_11S	expression vector for purification of 11S	[28]
pQE70	*E. coli* expression vector	Qiagen
pQE70-_Tstrep_SACOL2603_myc_−2602-2601_HA_-_his_TspA	for production of TspA with an N-terminal his tag, alongside LapT1 with an N-terminal twinstrep tag, LapT2 with an N-terminal myc tag and SACOL2601 with a C-terminal HA tag	this work

### 2.2. NanoLuc secretion assays

Furimazine was purchased from Promega (N1610) and used according to the manufacturer’s instructions. The NanoBit large subunit 11S was purified from *E. coli* BL21(DE3) [pBAD−_6H_11S] using the published protocol [28] and was used at a final concentration of 5 μM. For assay of 5 ml cultures, *S. aureus* strain COL was cultured as described earlier. Once cultures had reached a density of 0.5 (OD_600_), anhydrotetracycline (ATC; 250 ng ml^−1^) was added to induce expression of EsxA-pep86. Cells were harvested at a density of 2 (OD_600_) by centrifugation at 16 000 × *g*, 20°C. Cell pellets were re-suspended in TBS + 50 μg ml^−1^ lysostaphin (LSPN, Ambi) and incubated for 10 min at 37°C; 100 μl of each sample was supplemented with 5 µM 11S and 2 µl furimazine (1 : 100 dilution of stock solution) in a 96-well plate (Greiner, REF655073) with an approximate final well volume of 105 μl. Luminescence at 460 nm was read 3 min after incubation of the plate at room temperature, with a FLUOstar Omega using a gain value of 3000. Samples from the same cultures were prepared for immunoblot and analysed with antibodies as previously described [24]. For assays in 384-well plates, overnight *S. aureus* cultures were subcultured in fresh TSB at a density (OD_600_) of 0.001 for assay of EsxA-pep86 secretion or 0.01 for assay of TspA_NT_-pep86 secretion and cultured at 37°C/34°C/30°C with shaking at 200 rpm in a 384-well plate (Greiner, REF781098), with a well volume of 50 μl. In general, for each assay, one row of the 384-well plate was used (16 wells, i.e. 16 technical replicates). This was repeated in triplicate with three separate starter cultures (i.e. three biological replicates). The exceptions to this are in figure 2*d*,*e*, where in figure 2*d*, there is one biological replicate but 64 technical replicates and figure 2*e* where there are three biological repeats each of 64 technical replicates. For COL strains grown at 37°C, 250 ng ml^−1^ ATC was added after 320 min and 11S and furimazine was added after 380 min. For all other strains, 500 ng ml^−1^ ATC was added after 180 min (37°C cultures), 220 min (34°C cultures) or 240 min (30°C cultures). 11S and furimazine were added at 220 min (37°C cultures), 280 min (34°C cultures) or 300 min (30°C cultures). OD_600_ and luminescence readings were taken every 10 min. To measure total luminescence (i.e. combined extracellular and cytoplasmic levels), 50 μg ml^−1^ lysostaphin in TBS was added to each well and incubated for 10 min at 37°C. Luminescence readings were taken after supplementation of 11S and furimazine.

### 2.3. Cell fractionation and western blot

Single colonies of *S. aureus* harbouring the appropriate pRab11-based plasmid were cultured in TSB medium with chloramphenicol. On the following day, 600 µl of overnight culture was subcultured into 10 ml of fresh TSB. When the culture reached OD_600nm_ = 0.5–0.6, 500 ng ml^−1^ of ATC was added to induce plasmid-encoded gene expression. Cells were harvested at OD_600_ = 2. A 1.8 ml aliquot of the culture supernatant was retained, filtered through a 0.22 µm sterile filter and the filtrate supplemented with 50 µg ml^−1^ sodium deoxycholate (Merck, 89904) and 10% trichloroacetic acid (TCA; Cambridge Bioscience, 700016-10 ml-CAY). Proteins were precipitated on ice overnight and subsequently pelleted by centrifugation. After washing with 80% ice-cold ethanol, the pellet was re-suspended in re-suspension buffer (50 mM Tris–HCl, pH 8.0, 4% SDS, 10% glycerol) and then boiled with one-third volume of 4 × Laemmli sample buffer (Bio-Rad, 1610747) for 10 min. This was taken as the supernatant fraction. The harvested cells were re-suspended in Tris-buffered saline containing 100 µg ml^−1^ lysostaphin and incubated at 37°C for 30 min. This was then supplemented with one-third volume of 4 × Laemmli sample buffer and boiled for 10 min. This was taken as the whole cell fraction.

For western blot analysis, samples were separated by SDS PAGE, transferred to nitrocellulose membrane using a Trans-Blot (Bio-Rad) with a Whatman paper soaked in transfer buffer composed of 25 mM Tris, 192 mM glycine pH 8.3 and 10% methanol and analysed by incubation with one of the following primary antibodies: anti-EsxA [8], anti-TrxA [39], anti-HA tag (Merck, H9658), anti-strep tag (Qiagen, 34850), anti-6*his tag (Thermo Fisher Scientific,11533923), anti-myc tag (Abcam, Ab23) and an HRP-linked secondary antibody. A goat anti-mouse antibody (Bio-Rad, 1706516) was used with primary antibodies against the strep, myc and HA tags. A goat anti-rabbit antibody (Bio-Rad, 1721019) was used with all other primary antibodies. All uncropped western blots are provided in the electronic supplementary material.

### 2.4. Protein purification

LB medium containing ampicillin (0.125 mg ml^−1^) was inoculated 1/100 with an overnight pre-culture of strain M15 strain carrying pQE70-_Tstrep_SACOL2603-_myc_2602−2601_HA_-_his_TspA and grown at 37°C for 2 to 3 h with aeration, until OD_600_ reached approx. 0.5. Cultures were then supplemented with 0.5 mM isopropyl β-d-1-thiogalactopyranoside (IPTG), grown at 18°C overnight and then harvested by centrifugation. Cell pellets were re-suspended in binding buffer W (100 mM Tris–HCl, pH 8.0, 150 mM NaCl and 1 mM EDTA). Cells were lysed by sonication, the resulting cell lysate was clarified by centrifugation at 20 000*g* for 30 min, the supernatant was applied to a 1 ml Strep-tactin XT column (IBA, 24021-001), washed with buffer W (100 mM Tris–HCl, pH 8.0, 150 mM NaCl and 1 mM EDTA), and the bound proteins were eluted in buffer BXT (IBA, 2-1042-025). Proteins eluted from the Strep-tactin XT column were concentrated using a 50 MWCO Vivaspin centrifugal concentrator (Global Life Sciences Solutions Operations, 28932318) before injection onto a Superdex S200 10/300 GL column (Cytiva) equilibrated in 50 mM HEPES, pH 7.5 and 150 mM NaCl. Samples were analysed by SDS PAGE using Mini-PROTEAN^®^ TGX™ Precast Gels (Bio-Rad).

### 2.5. Other methods

Secretion data were analysed and presented using Prism (GraphPad). Raw data are available for download from FigShare (https://doi.org/10.6084/m9.figshare.25368598). Gene neighbourhood diagrams were generated using clinker (https://cagecat.bioinformatics.nl [40]).

## 3. Results

### 3.1. A NanoLuc assay for measuring type VII-dependent secretion in *S*. *aureus*

Previous methods to monitor *S. aureus* T7SSb secretion activity have relied almost exclusively on western blotting. Assessing secretion of endogenous WXG100-family proteins, in particular EsxA, involves isolating and concentrating culture supernatants, usually by precipitation with TCA [6,8,19,34]. Furthermore, detection of natively produced LXG toxins is generally too low to detect by western blotting even from concentrated supernatant samples, and assessing their secretion has required overproduction of epitope-tagged variants from an inducible plasmid [22–25]. These methods depend on the availability of good quality antibodies, are often poorly reproducible and are difficult to quantify.

To circumvent these issues, we utilized NanoBit to develop a new assay to monitor T7 secretion. NanoLuc is an engineered variant of a deep sea shrimp luciferase protein. It is relatively small (19 kDa) and has superior biophysical characteristics compared to other luciferases [41]. NanoBit is a complementation reporter system based on NanoLuc, which has previously been used to investigate protein secretion via the Sec and Tat translocons in *E. coli* [28,42,43]. Two separate NanoBit subunits (11S, a 156 amino acid large subunit, and pep86, an 11 amino acid small subunit) combine with high affinity to generate the functional enzyme, which luminesces with high intensity at 460 nm in the presence of the substrate furimazine and molecular oxygen [41].

**Figure 1.**
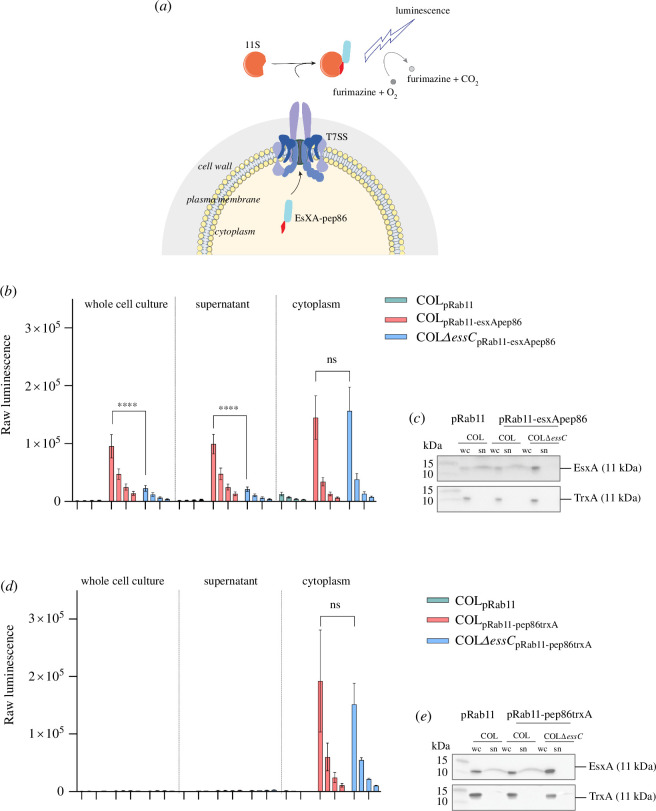
The NanoBit T7 secretion assay. (*a*) Schematic representation of the NanoBit secretion assay. The T7SSb substrate EsxA with a C-terminal pep86 tag is produced in the *S. aureus* cytoplasm, and 11S and furimazine (the NanoLuc large subunit and substrate) are provided outside the cell. Secretion of EsxA-pep86 via the T7SS results in functional complementation of 11S, which luminesces in the presence of furimazine and O_2_. Note that the cartoon is not an accurate representation of the stoichiometry and architecture of the T7SSb, which are unknown. (*b,d*) COL wild-type or Δ*essC* strains carrying the indicated plasmids were grown in 5 ml cultures. At OD_600_ = 0.5, ATC was added to induce gene expression from pRab11. When cells reached OD_600_ = 2, samples corresponding to the whole cell culture, the clarified culture medium (‘supernatant’) and lysed cells (‘cytoplasm’) were prepared. Serial twofold dilutions of whole cell cultures, clarified supernatant and cytoplasmic fractions were prepared, and 11S and furimazine were added to each. The data presented correspond to the mean luminescence readings of five biological replicates, and error bars represent the s.e.m. Two-way ANOVA was performed to determine statistical significance (ns *p* > 0.05; ****p < 0.0001). (*c,e*) Lysed whole cell (wc) and clarified culture supernatant (sn) fractions from (*b,d*) were analysed by immunoblot with antibodies against EsxA (top) or TrxA (bottom). Supernatant samples were concentrated by TCA precipitation prior to analysis, and the amount analysed corresponds to 6× more culture volume than for lysed cell samples.

We first selected EsxA as our model substrate (figure 1*a*). EsxA is produced at significantly higher copy than other T7SS components [8] and can be detected by western blot in culture supernatants of the *essC1* strain COL relatively reliably, allowing us to correlate our new assay with data from traditional immunoblot assays. We constructed plasmid pRab11-esxApep86 that permits inducible overproduction of EsxA with the NanoBit small subunit, pep86, fused to its C-terminus. This plasmid was transferred into wild-type and Δ*essC* variants of COL, and the wild-type strain was also transformed with empty pRab11 as a further control. Cultures (5 ml volume in 50 ml falcon tubes) were supplemented with ATC and grown at 37°C to mid-logarithmic phase (OD_600_ ≅ 2.0; for growth curves, see electronic supplementary material, figure S1). At this point, samples of whole cell culture, culture supernatant and lysostaphin-lysed cells were prepared. The NanoBit large subunit, 11S, and the NanoLuc substrate furimazine were added to serial twofold dilutions of these samples, and luminescence readings were taken (figure 1*b*). Cell lysate and culture supernatant samples from the same cultures were also analysed by immunoblot (figure 1*c*).

Luminescence was detected in all samples except those from cells carrying empty vector, signifying an EsxA-pep86-specific luminescence signal (figure 1*b*). Similar luminescence readings were obtained for both the whole cell culture and the supernatant samples, demonstrating that external pep86 can be measured without centrifugal separation of culture supernatants (figure 1*b*). Deletion of *essC* resulted in a dramatic reduction in the level of extracellular EsxA-pep86 compared to the wild-type strain, while the cytoplasmic level was similar in both strains. This confirms that the extracellular EsxA-pep86-dependent luminescence signal is due to secretion by the T7SS. We assign that residual low level of extracellular EsxA-pep86 in the *essC* mutant strain to cell leakage/lysis, or possibly secretion via an alternative pathway.

**Figure 2.**
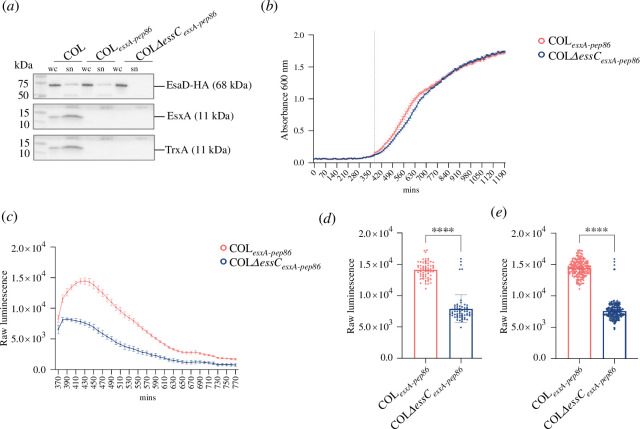
High-throughput secretion analysis of strains producing chromosomally encoded EsxA-pep86. (*a*) The indicated strains were grown in 5 ml cultures, and ATC was added at OD_600_ = 0.5 to induce gene expression from pRab11. ‘pEsaD-HA’ denotes plasmid pRab11-CBED-DEG, carrying an HA-tagged copy of the T7 substrate EsaD, along with its accessory secretion factors [24]. At OD_600_ = 2, lysed whole cell (wc) and clarified supernatant (sn) fractions were prepared and analysed by immunoblot with antibodies against the HA tag (to detect EsaD-HA), EsxA or TrxA. Supernatant samples were concentrated by TCA precipitation prior to analysis, and the amount analysed corresponds to 6× more culture volume than for lysed cell samples. (*b*) Growth curve for cultures of COL wild-type and Δ*essC* strains, each producing chromosomally encoded EsxA with a C-terminal pep86 tag from the native locus; 192 replicate 50 μl cultures (64 technical replicates × 3 biological replicates) were grown in 384-well plates at 37°C. Furimazine and 11S were added at 360 min (dotted line). Mean OD_600_ values are plotted, and error bars correspond to the s.e.m. (*n* = 3). (*c*) Luminescence readings at 10 min intervals for the cultures in (*b*) following initiation of luminescence with furimazine and 11S at 360 min. Error bars correspond to the s.e.m. (*n* = 3). (*d*) A single overnight culture of each COL*_esxA-pep86_* and COLΔ*essC_esxA-pep86_* was diluted to an OD_600_ of 0.1 and used to inoculate 64 individual wells of a 384-well plate. After 360 min growth, furimazine and 11S were added and a single luminescence reading from each well was taken at 430 min. (*e*) As for (*d*) except that three overnight cultures of each COL*_esxA-pep86_* and COL Δ*essC_esxA-pep86_* were used, giving 192 individual data points for each strain. Statistical significance in (*d*,*e*) was assessed with the Mann–Whitney test, with **** indicating *p* < 0.0001 for each case.

When fractions of the same cultures were analysed by immunoblot, a single band at approximately 11 kDa was detected with antibodies against EsxA in all three strains (figure 1*c*), regardless of whether the cells harboured pRab11 or pRab11-esxApep86. Since two of the three strains analysed produce both endogenous chromosomally encoded EsxA (11 kDa) and plasmid-encoded EsxA-pep86 (12.3 kDa), we infer that this 11 kDa band corresponds to endogenous EsxA and that the pep86 fusion prevents detection by the EsxA antibodies (refer also to figure 2*c* where chromosomal tagging of *esxA* with pep86 prevents antibody detection).

As an additional control, we constructed a similar fusion of a cytoplasmic protein, TrxA, with an N-terminal pep86 tag (pRab11-pep86-TrxA) and assayed this in the same way. Luminescence readings showed that while pep86-TrxA was produced, barely any signal was detected outside the cells (figure 1*d*). Immunoblots confirmed the cytoplasmic localization of TrxA and showed that EsxA was secreted normally in these strains, although similarly to EsxA-pep86, the pep86-TrxA fusion was not detected by our TrxA antibodies (figure 1*e*). In summary, figure 1 demonstrates that luminescence readings correlate with T7-dependent secretion of EsxA-pep86 and that extracellular EsxA-pep86 can be measured in unprocessed whole cell cultures.

### 3.2. High-throughput analysis of EsxA secretion

Having established that we can use the NanoBit assay to measure T7SS secretion in *S. aureus* and that we can directly detect extracellular EsxA-pep86 from growing cultures, we next sought to miniaturize it for high-throughput format to improve quantitation and sensitivity. To this end, we engineered a C-terminal pep86 fusion onto chromosomally encoded EsxA, to avoid the need for an inducible plasmid. This ensures that the assay is undertaken using a system that is as close to native as possible. To assess whether the chromosomally encoded EsxA-pep86 fusion was competent to support T7 secretion, we transformed this strain with a plasmid encoding a HA-tagged copy of the T7 LXG toxin substrate EsaD alongside its accessory proteins. Immunoblot data for cell lysate and culture supernatant samples (figure 2*a*) showed that although the EsxA-pep86 fusion was not detected by our antibodies, as we had seen previously with the plasmid-encoded fusion, secretion of EsaD-HA was unaffected by the presence of the pep86 tag on EsxA (figure 2*a*). In a separate experiment, we confirmed that EsaD-HA secretion is indeed dependent on EsxA in *essC1* strains (electronic supplementary material, figure S2). We therefore conclude that EsxA-pep86 is functional.

We next performed growth curves for 50 μl cultures of COL*_esxApep86_* and an otherwise isogenic *essC* mutant in the 384-well plate format. Strains grew slowly under these conditions, with cultures reaching the stationary phase approximately 20 h (1200 min) after inoculation at an OD_600_ of 0.001 (figure 2*b*). The *essC* mutant strain grew slightly more slowly in the exponential phase but reached the same final OD_600_ as the *essC* ^+^ strain. The half-life of the NanoLuc luminescence signal is reported to be approx. 120 min (Promega) meaning that continuous monitoring of secreted EsxA-pep86 throughout exponential growth would not be possible. We therefore chose to monitor secretion during early exponential phase, and to this end, furimazine and 11S were added to cultures at the beginning of exponential phase, after 6 h (360 min) growth (figure 2*b*, dotted line) to initiate luminescence. Luminescence readings were then taken at 10 min intervals. The luminescence signal for COL*_esxApep86_* cultures initially rose, peaking 70 min after initiation of luminescence, indicating that cells were actively secreting protein during this time (figure 2*c*). The subsequent decline in luminescence intensity is likely due to signal decay, although degradation of pep86 might also contribute. As seen in the previous assay shown in figure 1, some extracellular EsxA-pep86 was detected in the Δ*essC* strain (figure 2*c*).

To analyse the distribution of secretion activity within a clonal population, a single colony of each of COL*_esxApep86_* and COLΔ*essC_esxApep86_* was used to inoculate overnight cultures, and these were each used to inoculate 64 × 50 μl cultures and assayed as described earlier. Luminescence readings taken at 430 min (OD_600_ = 0.23) were used to generate a scatter plot of individual data points (figure 2*d*). This analysis reveals a moderate distribution of secretion activity in replicate cultures of COL*_esxApep86_*; however, the distribution of extracellular EsxA-pep86 levels in the control COLΔ*essC_esxApep86_* strain was quite broad, overlapping the *essC* ^+^ strain and highlighting the requirement for a high-throughput assay. The same analysis was repeated for three independent cultures of each strain, and this did not increase variability (figure 2*e*).

### 3.3. Variable secretion characteristics of different *S. aureus* strains

**Figure 3.**
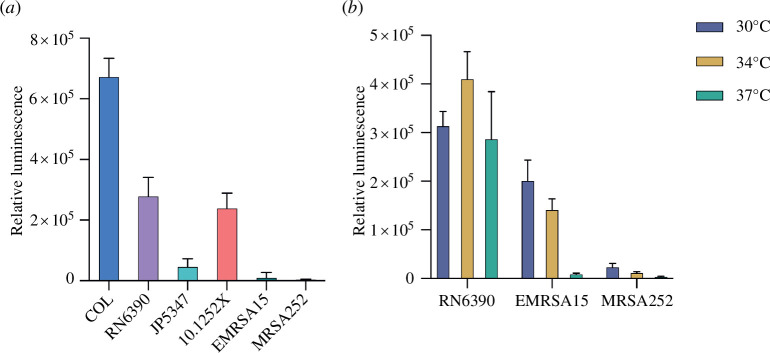
EsxA secretion in different *S. aureus* strains and at different temperatures. (*a*) Wild-type and Δ*essC* variants of the indicated strains were transformed with pRab11-esxApep86 and cultured in 384-well plates at 37°C. Expression of *esxA-pep86* was induced by the addition of ATC at 180 min, and luminescence was initiated by the addition of furimazine and 11S at 220 min. Luminescence readings taken at 10 min intervals (shown in electronic supplementary material, figure S1*b*) were divided by OD_600_ readings to give the relative luminescence. The values for Δ*essC* relative luminescence were subtracted from those of the wild-type relative luminescence to give a relative secretion value for each time point. For each strain pair, the time point with the peak value for relative secretion is presented. Data represent the mean of three biological replicates each with 16 technical replicates. Error bars correspond to the s.e.m. (*n* = 3). (*b*) Secretion assays were performed as in (*a*), but each strain pair was cultured at 30, 34 and 37°C (data for 37°C are reproduced from (*a*) for the sake of comparison). The timings of ATC induction and luminescence initiation were adjusted for the slower growth rates at 30 and 34°C (electronic supplementary material, S2A, and §2), and raw luminescence readings are shown in electronic supplementary material, figure S2*b*.

It has previously been reported that T7SS activity varies across different *S. aureus* isolates and growth conditions [8,19,34]. However, direct comparison across different *S. aureus* strains has been hampered by low secretion activity (often below the detection limit) and low reproducibility (our unpublished observations). We therefore used the NanoBit assay to compare secretion across cell cultures of five additional *S. aureus* isolates, two of which (like COL) carry the *essC1* variant *ess* locus (RN6390 and JP5347) and one representative isolate for each of *essC2* (10.125.2X), *essC3* (MRSA252) and *essC4* (EMRSA15). Each strain, along with its otherwise isogenic Δ*essC* mutant, was transformed with pRab11-esxApep86 and assayed for EsxA-pep86 secretion in 384-well plates. Growth curves showed that each Δ*essC* strain grew similarly to its parental strain (electronic supplementary material, figure S3*a*). The five additional strains tested here all entered exponential phase faster than COL, and so the timing of addition of ATC (to induce EsxA-pep86 production) and of 11S and furimazine was adjusted accordingly (§2). T7SS-dependent secretion of EsxA-pep86 was observed, but to varying degree, in the three *essC1* isolates and the *essC2* strain 10.125.2X. However, no T7-secreted EsxA could be detected in the *essC3* (MRSA252) and *essC4* (EMRSA-15) strains (figure 3*a*; electronic supplementary material, figure S3*b*). To confirm that EsxA-pep86 was being produced in all of the strains, we measured total luminescence in each culture following in-well cellular lysis (a combined measure of cytoplasmic and secreted EsxA-pep86 levels) (electronic supplementary material, figure S3*c*). Substantive luminescence could be detected even in lysed cultures of MRSA252 and EMRSA-15, indicating that these strains produce but do not secrete EsxA-pep86 by the type VII pathway. For comparison, we also used the traditional immunoblot method to assay secretion of endogenous EsxA in the same strains and obtained similar results but with less sensitivity (electronic supplementary material, figure S3*d*).

**Figure 4.**
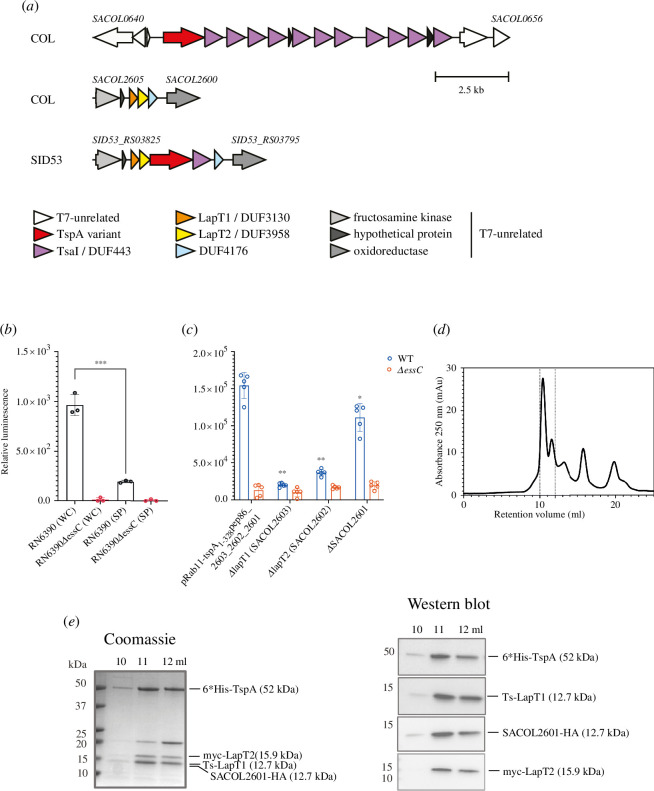
Identification of TspA secretion factors. (*a*) Locus diagrams depicting the genetic neighbourhood of *tspA* (*SACOL0643*, red—top) and the T7SS-associated genes *SACOL2603*, *SACOL2602* and *SACOL2601* (orange, yellow and blue—middle) in strain COL. Bottom—the *SACOL2603* equivalent locus in strain SID53 has a *tspA2-tsaI2* insertion between the homologues of *SACOL2602* and *SACOL2601. SACOL2603* encodes a DUF3130/TIGR04917 family protein. (*b*) COL wild-type or Δ*essC* strains carrying plasmid pRab11-tspA1-328-pep86_2603_2602_2601 were grown in 5 ml cultures at 37°C. At OD_600_ = 0.5, ATC was added to induce gene expression from pRab11. When cells reached OD_600_ = 2, samples corresponding to the whole cell culture (WC), the clarified culture medium (SP) were prepared and 11S and furimazine were added to each. The data presented correspond to the mean luminescence readings of three biological replicates, and error bars represent the s.e.m. Statistical significance for whole cell culture of the wild-type versus clarified culture medium was assessed with the Mann–Whitney test, with *** indicating *p* < 0.001. There was no significant difference between values for the whole cell versus clarified culture medium for the Δ*essC* strain. (*c*) Wild-type and Δ*essC* variants of strain RN6390 were transformed with the indicated plasmids and cultured in 384-well plates at 34°C. Expression from pRab11 was induced by the addition of ATC at 120 min, and luminescence was initiated by the addition of furimazine and 11S at 260 min. Luminescence readings were taken at the 280 min time point and were divided by OD_600_ readings to give the relative luminescence. Data represent the mean of five biological replicates each with 16 technical replicates. Error bars correspond to the s.e.m. (*n* = 5). The relative luminescence of RN6390 harbouring each plasmid containing the indicated individual gene deletion was compared with the relative luminescence signal of RN6390 harbouring pRab11-tspA_1-328_-pep86_2603_2602_2601 using the Mann–Whitney test, *p < 0.05; **p < 0.01. (*d*) *Escherichia coli* M15 cells carrying pQE70-_Tstrep_SACOL2603_myc_-2602-2601_HA_-_his_TspA were grown in LB medium. Following induction of gene expression with IPTG, complexes were purified by streptactin-affinity purification, and the resulting sample was analysed by size exclusion chromatography (SEC). (*e*) Fractions from within the dotted lines in (*d*) were subject to SDS-PAGE and analysed by Coomassie staining (left panel) or by western blotting with antibodies against the tags on each component (right panel).

Since no T7SS activity was detected for strains MRSA252 and EMRSA15 grown at 37°C, we wondered whether these isolates might exhibit a different temperature dependence for secretion. We therefore repeated the NanoBit assay with these strains, alongside RN6390 as an *essC1* control, using cell cultures growing at 30 and 34°C (figure 3*b* and electronic supplementary material, S4). For EMRSA15, EsxA-pep86 was secreted at both of the lower temperatures, while for MRSA252, the lower temperatures made little difference to secretion activity, which was negligible at all temperatures tested. EsxA was secreted similarly at all three temperatures in RN6390 (figure 3 and electronic supplementary material, S4). Broadly similar results were obtained from immunoblot assay of endogenous EsxA secretion, although some variability was observed, highlighting a limitation of this method (electronic supplementary material, figure S4*d*). Temperature therefore contributes to the regulation of T7SS activity in *S. aureus* in a strain-specific manner. Of note, EsxA-pep86 leakage was markedly lower at 30 and 34°C than at 37°C in each of the Δ*essC* strains (electronic supplementary material, figure S4*b*).

### 3.4. Identification of TspA secretion partners

TspA is a membrane-depolarizing LXG toxin found across all *S. aureus essC* variant strains. It is encoded at a locus remote from the T7SS structural genes and precedes a string of non-identical genes coding for DUF443 membrane proteins, which provide protection against TspA activity [22] (figure 4*a*). Recent analysis of other T7SSb LXG toxins has revealed a common mechanistic feature for secretion, involving formation of a hetero-oligomeric pre-secretion complex between the LXG domain and helical hairpin partner proteins encoded at the same genetic locus as the toxin [24,26,27,44]. Logically, the TspA LXG domain should also be expected to require similar helical binding partners to generate a secretion-competent structure. However, there are no candidate genes coding for predicted helical proteins in the immediate neighbourhood of *tspA* (*SACOL0643*) (figure 4*a*).

Genomic analysis previously identified a conserved module of three small genes, potentially implicated in T7 secretion, at a locus distant from both the *tspA* and *ess* gene clusters [4] (figure 4*a*, shaded orange, yellow and blue). In a handful of *S. aureus* strains, e.g. SID53, a second homologue of *tspA* is present at this locus, along with a DUF443-encoding immunity gene (figure 4*a*). However, in most well-studied strains, including COL, USA300 and RN6390, there is no *tspA* homologue or immunity gene at this locus, although the small genes are always present. This raised the possibility that the conserved cluster may be required for the biogenesis of TspA. Analysis of the encoded proteins indicates that SACOL2603 and SACOL2602 are predicted to be helical hairpin proteins belonging to the Lap1 (DUF3130) and Lap2 (DUF3958) families of WXG100-like export factors, respectively. Members of these protein families have been characterized in *Streptococcus intermedius* and shown to interact with the LXG domain of the lipid II phosphatase toxin, TelC [11,26]. Following the nomenclature developed in *S. intermedius* and based on results described below, we have renamed SACOL2603 and SACOL2602 as LapT1 and LapT2, respectively (for LXG-associated α-helical protein for TspA). The third protein encoded at this locus, SACOL2601, has a DUF4176 domain. Genes encoding DUF4176 domains are frequently associated with T7SS loci [3], and the involvement of a DUF4176 domain protein in LXG toxin secretion was recently determined in *S. intermedius* [27].

We therefore adapted the NanoBit assay to test the prediction that LapT1, LapT2 and SACOL2601 function in TspA secretion. We constructed a plasmid carrying *tspA_1–328_-pep86*, *SACOL2603*/*lapT1, SACOL2602*/*lapT2* and *SACOL2601*, avoiding issues arising from TspA toxicity by using a truncated version lacking the toxin domain but supplied with a C-terminal pep86 tag. Owing to the slow growth kinetics of strain COL in 384-well plates, we carried out assays in RN6390, whose sequence is identical to that of COL at the *tspA* and *SACOL2603* loci, except for a single (synonymous) nucleotide change in *tspA*. Following our observation of much lower EsxA-pep86 leakage into the supernatant at lower temperatures, we opted to conduct the secretion assays at 34°C.

After introduction of the plasmid into RN6390 wild-type and Δ*essC* strains, we measured luminescence in whole cell cultures and in clarified culture supernatant following pelleting of cells (figure 4*b*). The presence of pep86-tagged TspA_NT_ in whole cell cultures was clearly detected, dependent on an active T7SS. However, little or no TspA_NT_ was detected in the fractionated culture supernatant, confirming the previous finding that TspA is cell surface associated rather than fully secreted [22]. These results also indicate that the TspA toxin domain (residues 329–469) is dispensable for cell surface attachment.

To determine whether any of LapT1, LapT2 and SACOL2601 are required to support secretion of TspA, we individually deleted each of the encoding genes from the plasmid and used the high-throughput assay in whole cell cultures to assess export of pep86-tagged TspA_NT_. Figure 4*c* indicates that in the absence of each gene, significantly less TspA_NT_-pep86 could be detected in whole cell cultures, while the cytoplasmic levels (deduced from measuring total luminescence per well following lysis) were not much affected (electronic supplementary material, S5B). We conclude that each of LapT1, LapT2 and SACOL2601 supports TspA secretion. It should be noted that the chromosomal copy of the *SACOL2601−2603* gene cluster is present in these strains and may account for some of the residual secretion seen from the plasmid deletion constructs.

Previous work has reported that LXG proteins form a stable pre-secretion complex with their cognate helical hairpin secretion partners [23,24,26,27]. To investigate whether any of LapT1, LapT2 or SACOL2601 interacts with TspA, we co-produced all four proteins in *E. coli*. The recombinant proteins carried an N-terminal twinstrep tag on LapT1 for purification and a C-terminal HA, N-terminal myc and N-terminal his tag on SACOL2601, LapT2 and TspA, respectively, for detection. Complexes were purified by streptactin affinity chromatography and analysed by size exclusion chromatography (SEC; figure 4*d*) and both SDS PAGE and western blotting (figure 4*e*). All four proteins co-eluted in the SEC main peak confirming that they exist as a tetrameric complex. Taken together, our results indicate that the *SACOL2601−2603* gene cluster is required for the biogenesis of TspA.

## 4. Discussion

Here, we report a novel, sensitive assay for monitoring protein secretion by the T7SS in *S. aureus*, using the NanoBit split luciferase system. The 11 amino acid pep86 fragment of NanoBit is small enough to be compatible with secretion when fused to the WXG100 protein EsxA or to a larger LXG protein substrate. As the presence of luminescence from secreted proteins can be measured directly in unprocessed cell cultures, this enables high-throughput screening of large numbers of strains and growth conditions.

Most studies of the *S. aureus* T7SS to date have focused on secretion by *essC1* strains, using a handful of isolates. Strains exhibit high genetic diversity at their T7 loci [14], but experimental investigation of *essC2* to *essC4* variant strains has previously been constrained by low activity in laboratory conditions and the lack of a sensitive secretion assay [34]. Using the NanoBit assay, we found substantial variability in the secretion efficiencies of such strains and their temperature dependence. In particular, the EMRSA15 T7SS (an *essC4* variant strain), which was inactive at 37°C, exhibited a significant level of secretion at 30 and 34°C, suggesting a role for temperature in T7SSb regulation. Interestingly, *ess* transcription and T7 secretion were previously shown to be activated by reducing membrane fluidity, which was achieved by treating cells with *cis*-unsaturated fatty acids [45,46]. Temperature-induced changes in membrane fluidity may potentially account for the differences in secretion observed here.

A number of pathways have been implicated in transcriptional regulation of the *S. aureus* T7SS, including the SaeRS and ArlRS two-component systems, the Agr quorum-sensing system and the alternative sigma factor SigB [19,45,47–50]. However, the complex regulatory picture is far from complete and key questions around how, why and when the secretion system is expressed remain unanswered. Moreover, additional factors may activate secretion at the post-transcriptional level, e.g. the presence of haemin has been linked to post-transcriptional activation of the T7SS in some strains [34,51]. The availability of a high-throughput assay will facilitate screening of a range of conditions, paving the way for a much more detailed analysis of T7 regulation than has been possible to date.

We employed the NanoBit assay to explore the secretion of the surface-attached LXG toxin, TspA. While other LXG toxins characterized to date are encoded alongside accessory proteins essential for their biogenesis [24,26,27], secretion partners for TspA had not been identified. A conserved cluster of three genes remote from the *tspA* locus had previously been bioinformatically linked to the T7SS [4]. We showed that each of these genes supports secretion of a TspA-pep86 fusion, suggesting that their genomic conservation is because they are required for TspA biogenesis. This conclusion was further supported by co-purification experiments, where tagged variants of the three proteins formed a complex with TspA. Two of these genes, *SACOL2603* and *SACOL2602*, code for helical hairpin proteins of the DUF3130 and DUF3958 families, respectively. The LXG toxins, TelC and TelD, from *S. intermedius* both require proteins from each of these families for secretion, with Lap1 being a DUF3130 protein and Lap2 a DUF3958 protein. In each case, the Lap1 and Lap2 proteins were shown to interact with the LXG domain of their cognate toxin partners. In keeping with this nomenclature, we have renamed SACOL2603 as LapT1 and SACOL2602 as LapT2. Klein *et al*. [26] identified a conserved FxxxD motif close to the C-terminus of LapD1, showing that individual alanine substitutions of either conserved residue were sufficient to prevent TelD secretion. LapD1 and LapT1 share only limited sequence similarity; however, the FxxxD motif is present at the same position in both proteins (electronic supplementary material, figure S6). This motif is conserved across LapT1 homologues in other staphylococci and in enterococci and is likely to serve as a secretion motif for TspA export.

The third protein gene, *SACOL2601*, codes for a DUF4176 protein. Interestingly, proteins of this family are also encoded in the *S. intermedius telA*, *telB* and *telD* gene clusters, but not *telC*. While the homologue encoded at the *telA* locus was shown to be essential for TelA secretion, it was not required for TelC export [27]. It is not clear whether TelC requires a DUF4176 protein encoded by a different cluster or whether its secretion occurs independently of such a protein. In addition to TspA, a variant-specific LXG protein-coding gene is found at the *ess* loci of *essC1*, essC*2*, *essC3* and *essC4 S. aureus* strains [4]. However, no DUF4176-encoding genes are found at any *ess* loci, suggesting either that they are not universally required or that a single DUF4176 protein might function in secretion of multiple substrates. Further work would be required to investigate this.

In conclusion, we describe a novel, sensitive assay to assess secretion of T7 substrate proteins in *S. aureus*. We anticipate that this approach should be broadly applicable for the study of type VII secretion across a range of organisms.

## Data Availability

The datasets supporting this article have been uploaded to Figshare [52]. Supplementary material is available online [53].

## References

[1] Turner NA , Sharma-Kuinkel BK , Maskarinec SA , Eichenberger EM , Shah PP , Carugati M , Holland TL , Fowler VG . 2019 Methicillin-resistant Staphylococcus aureus: an overview of basic and clinical research. Nat. Rev. Microbiol. **17** , 203–218. (10.1038/s41579-018-0147-4) 30737488 PMC6939889

[2] Shariati A , Dadashi M , Moghadam MT , van Belkum A , Yaslianifard S , Darban-Sarokhalil D . 2020 Global prevalence and distribution of vancomycin resistant, vancomycin intermediate and heterogeneously vancomycin intermediate Staphylococcus aureus clinical isolates: a systematic review and meta-analysis. Sci. Rep. **10** , 12689. (10.1038/s41598-020-69058-z) 32728110 PMC7391782

[3] Boardman ER , Palmer T , Alcock F . 2023 Interbacterial competition mediated by the type VIIb secretion system. Microbiol **169** , 001420. (10.1099/mic.0.001420) PMC10765036 38116759

[4] Bowman L , Palmer T . 2021 The type VII secretion system of Staphylococcus. Annu. Rev. Microbiol. **75** , 471–494. (10.1146/annurev-micro-012721-123600) 34343022 PMC7619418

[5] Burts ML , DeDent AC , Missiakas DM . 2008 EsaC substrate for the ESAT-6 secretion pathway and its role in persistent infections of Staphylococcus aureus *.* Mol. Microbiol. **69** , 736–746. (10.1111/j.1365-2958.2008.06324.x) 18554323 PMC2597432

[6] Burts ML , Williams WA , DeBord K , Missiakas DM . 2005 EsxA and esxb are secreted by an ESAT-6-like system that is required for the pathogenesis of Staphylococcus aureus infections. Proc. Natl Acad. Sci. USA **102** , 1169–1174. (10.1073/pnas.0405620102) 15657139 PMC545836

[7] Ishii K , Adachi T , Yasukawa J , Suzuki Y , Hamamoto H , Sekimizu K . 2014 Induction of virulence gene expression in Staphylococcus aureus by pulmonary surfactant. Infect. Immun. **82** , 1500–1510. (10.1128/IAI.01635-13) 24452679 PMC3993393

[8] Kneuper H *et al* . 2014 Heterogeneity in ess transcriptional organization and variable contribution of the ess/type VII protein secretion system to virulence across closely related Staphylocccus aureus strains. Mol. Microbiol. **93** , 928–943. (10.1111/mmi.12707) 25040609 PMC4285178

[9] Wang Y *et al* . 2016 Role of the ESAT-6 secretion system in virulence of the emerging community-associated Staphylococcus aureus lineage ST398. Sci. Rep. **6** , 25163. (10.1038/srep25163) 27112266 PMC4844983

[10] Garrett SR , Higginson AB , Palmer T . 2024 Multiple variants of the type VII secretion system in Gram-positive bacteria. microLife **5** , uqae013. (10.1093/femsml/uqae013) 38957458 PMC11217815

[11] Whitney JC *et al* . 2017 A broadly distributed toxin family mediates contact-dependent antagonism between Gram-positive bacteria. eLife **6** , e26938. (10.7554/eLife.26938) 28696203 PMC5555719

[12] Kobayashi K . 2021 Diverse LXG toxin and antitoxin systems specifically mediate intraspecies competition in Bacillus subtilis biofilms. PLoS Genet. **17** , e1009682. (10.1371/journal.pgen.1009682) 34280190 PMC8321402

[13] Chatterjee A , Willett JLE , Dunny GM , Duerkop BA . 2021 Phage infection and sub-lethal antibiotic exposure mediate Enterococcus faecalis type VII secretion system dependent inhibition of bystander bacteria. PLoS Genet. **17** , e1009204. (10.1371/journal.pgen.1009204) 33411815 PMC7790226

[14] Warne B , Harkins CP , Harris SR , Vatsiou A , Stanley-Wall N , Parkhill J , Peacock SJ , Palmer T , Holden MTG . 2016 The ess/type VII secretion system of Staphylococcus aureus shows unexpected genetic diversity. BMC Genom. **17** , 222. (10.1186/s12864-016-2426-7) PMC4788903 26969225

[15] Bunduc CM , Fahrenkamp D , Wald J , Ummels R , Bitter W , Houben ENG , Marlovits TC . 2021 Structure and dynamics of a mycobacterial type VII secretion system. Nature **593** , 445–448. (10.1038/s41586-021-03517-z) 33981042 PMC8131196

[16] Beckham KSH *et al* . 2021 Structure of the mycobacterial ESX-5 type VII secretion system pore complex. Sci. Adv. **7** , eabg9923. (10.1126/sciadv.abg9923) 34172453 PMC8232910

[17] Jäger F , Kneuper H , Palmer T . 2018 EssC is a specificity determinant for Staphylococcus aureus type VII secretion. Microbiology **164** , 816–820. (10.1099/mic.0.000650) 29620499 PMC5994694

[18] Huppert LA , Ramsdell TL , Chase MR , Sarracino DA , Fortune SM , Burton BM . 2014 The ESX system in Bacillus subtilis mediates protein secretion. PLoS One **9** , e96267. (10.1371/journal.pone.0096267) 24798022 PMC4010439

[19] Anderson M , Aly KA , Chen YH , Missiakas D . 2013 Secretion of atypical protein substrates by the ESAT-6 secretion system of Staphylococcus aureus. Mol. Microbiol. **90** , 734–743. (10.1111/mmi.12395) 24033479 PMC3951145

[20] Renshaw PS *et al* . 2005 Structure and function of the complex formed by the tuberculosis virulence factors CFP-10 and ESAT-6. EMBO J. **24** , 2491–2498. (10.1038/sj.emboj.7600732) 15973432 PMC1176459

[21] Pallen MJ . 2002 The ESAT-6/WXG100 superfamily—and a new Gram-positive secretion system? Trends Microbiol. **10** , 209–212. (10.1016/s0966-842x(02)02345-4) 11973144

[22] Ulhuq FR *et al* . 2020 A membrane-depolarizing toxin substrate of the Staphylococcus aureus type VII secretion system mediates intraspecies competition. Proc. Natl Acad. Sci. USA **117** , 20836–20847. (10.1073/pnas.2006110117) 32769205 PMC7456083

[23] Garrett SR *et al* . 2023 A type VII-secreted lipase toxin with reverse domain arrangement. Nat. Commun. **14** , 8438. (10.1038/s41467-023-44221-y) 38114483 PMC10730906

[24] Yang Y , Boardman E , Deme J , Alcock F , Lea S , Palmer T . 2023 Three small partner proteins facilitate the type VII-dependent secretion of an antibacterial nuclease. mBio **14** , e0210023. (10.1128/mbio.02100-23) 37815362 PMC10653861

[25] Cao Z , Casabona MG , Kneuper H , Chalmers JD , Palmer T . 2016 The type VII secretion system of Staphylococcus aureus secretes a nuclease toxin that targets competitor bacteria. Nat. Microbiol. **2** , 16183. (10.1038/nmicrobiol.2016.183) 27723728 PMC5325307

[26] Klein TA , Grebenc DW , Shah PY , McArthur OD , Dickson BH , Surette MG , Kim Y , Whitney JC . 2022 Dual targeting factors are required for LXG toxin export by the bacterial type VIIb secretion system. mBio **13** , e0213722. (10.1128/mbio.02137-22) 36036513 PMC9600955

[27] Klein TA , Shah PY , Gkragkopoulou P , Grebenc DW , Kim Y , Whitney JC . 2024 Structure of a tripartite protein complex that targets toxins to the type VII secretion system. Proc. Natl Acad. Sci. USA **121** , e2312455121. (10.1073/pnas.2312455121) 38194450 PMC10801868

[28] Pereira GC , Allen WJ , Watkins DW , Buddrus L , Noone D , Liu X , Richardson AP , Chacinska A , Collinson I . 2019 A high-resolution luminescent assay for rapid and continuous monitoring of protein translocation across biological membranes. J. Mol. Biol. **431** , 1689–1699. (10.1016/j.jmb.2019.03.007) 30878481 PMC6461198

[29] Dyke KG , Jevons MP , Parker MT . 1966 Penicillinase production and intrinsic resistance to penicillins in Staphylococcus aureus. Lancet **1** , 835–838. (10.1016/s0140-6736(66)90182-6) 4159958

[30] Gill SR *et al* . 2005 Insights on evolution of virulence and resistance from the complete genome analysis of an early methicillin-resistant Staphylococcus aureus strain and a biofilm-producing methicillin-resistant Staphylococcus epidermidis strain. J. Bacteriol. **187** , 2426–2438. (10.1128/JB.187.7.2426-2438.2005) 15774886 PMC1065214

[31] Novick RP , Ross HF , Projan SJ , Kornblum J , Kreiswirth B , Moghazeh S . 1993 Synthesis of Staphylococcal virulence factors is controlled by a regulatory RNA molecule. EMBO J. **12** , 3967–3975. (10.1002/j.1460-2075.1993.tb06074.x) 7691599 PMC413679

[32] Garrett SR , Mariano G , Palmer T . 2022 Genomic analysis of the progenitor strains of Staphylococcus aureus RN6390. Access Microbiol. **4** , 11. (10.1099/acmi.0.000464.v3) PMC9996129 36910860

[33] Holden MTG *et al* . 2004 Complete genomes of two clinical Staphylococcus aureus strains: evidence for the rapid evolution of virulence and drug resistance. Proc. Natl Acad. Sci. USA **101** , 9786–9791. (10.1073/pnas.0402521101) 15213324 PMC470752

[34] Casabona MG , Kneuper H , Alferes de Lima D , Harkins CP , Zoltner M , Hjerde E , Holden MTG , Palmer T . 2017 Haem-iron plays a key role in the regulation of the ess/type VII secretion system of Staphylococcus aureus RN6390. Microbiology **163** , 1839–1850. (10.1099/mic.0.000579) 29171824 PMC5845736

[35] Donker T *et al* . 2017 Population genetic structuring of methicillin-resistant Staphylococcus aureus clone EMRSA-15 within UK reflects patient referral patterns. Microb. Genom. **3** , e000113. (10.1099/mgen.0.000113) 29026654 PMC5605955

[36] Helle L , Kull M , Mayer S , Marincola G , Zelder ME , Goerke C , Wolz C , Bertram R . 2011 Vectors for improved Tet repressor-dependent gradual gene induction or silencing in Staphylococcus aureus. Microbiology **157** , 3314–3323. (10.1099/mic.0.052548-0) 21921101

[37] Monk IR , Shah IM , Xu M , Tan MW , Foster TJ . 2012 Transforming the untransformable: application of direct transformation to manipulate genetically Staphylococcus aureus and Staphylococcus epidermidis. mBio **3** , e00277-11. (10.1128/mBio.00277-11) 22434850 PMC3312211

[38] Monk IR , Stinear TP . 2021 From cloning to mutant in 5 days: rapid allelic exchange in Staphylococcus aureus. Access Microbiol. **3** , 000193. (10.1099/acmi.0.000193) 34151146 PMC8209637

[39] Miller M *et al* . 2010 Staphylococcal pknb as the first prokaryotic representative of the proline-directed kinases. PLoS One **5** , e9057. (10.1371/journal.pone.0009057) 20140229 PMC2816222

[40] Gilchrist CLM , Chooi YH . 2021 Clinker & clustermap.js: automatic generation of gene cluster comparison figures. Bioinformatics **37** , 2473–2475. (10.1093/bioinformatics/btab007) 33459763

[41] England CG , Ehlerding EB , Cai W . 2016 NanoLuc: a small luciferase is brightening up the field of bioluminescence. Bioconjug. Chem. **27** , 1175–1187. (10.1021/acs.bioconjchem.6b00112) 27045664 PMC4871753

[42] Allen WJ , Watkins DW , Dillingham MS , Collinson I . 2020 Refined measurement of secA-driven protein secretion reveals that translocation is indirectly coupled to ATP turnover. Proc. Natl Acad. Sci. USA **117** , 31808–31816. (10.1073/pnas.2010906117) 33257538 PMC7749344

[43] Zhou WJ , Hao BH , Bricker TM , Theg SM . 2023 A real-time analysis of protein transport via the twin arginine translocation pathway in response to different components of the protonmotive force. J. Biol. Chem. **299** , 105286. (10.1016/j.jbc.2023.105286) 37742925 PMC10641609

[44] Spencer BL *et al* . 2023 Heterogeneity of the group B streptococcal type VII secretion system and influence on colonization of the female genital tract. Mol. Microbiol. **120** , 258–275. (10.1111/mmi.15115) 37357823 PMC10527989

[45] Kenny JG , Ward D , Josefsson E , Jonsson IM , Hinds J , Rees HH , Lindsay JA , Tarkowski A , Horsburgh MJ . 2009 The response to unsaturated long chain free fatty acids: survival mechanisms and virulence implications. PLoS One **4** , e4344. (10.1371/journal.pone.0004344) 19183815 PMC2629846

[46] Lopez MS , Tan IS , Yan D , Kang J , McCreary M , Modrusan Z , Austin CD , Xu M , Brown EJ . 2017 Host-derived fatty acids activate type VII secretion in Staphylococcus aureus. Proc. Natl Acad. Sci. USA **114** , 11223–11228. (10.1073/pnas.1700627114) 28973946 PMC5651732

[47] Bischoff M , Entenza JM , Giachino P . 2001 Influence of a functional sigB operon on the global regulators sar and agr in Staphylococcus aureus. J. Bacteriol. **183** , 5171–5179. (10.1128/JB.183.17.5171-5179.2001) 11489871 PMC95394

[48] Crosby HA , Tiwari N , Kwiecinski JM , Xu Z , Dykstra A , Jenul C , Fuentes EJ , Horswill AR . 2020 The Staphylococcus aureus arlRS two-component system regulates virulence factor expression through MgrA. Mol. Microbiol. **113** , 103–122. (10.1111/mmi.14404) 31618469 PMC7175635

[49] Dunman PM *et al* . 2001 Transcription profiling-based identification of Staphylococcus aureus genes regulated by the agr and/or sarA loci. J. Bacteriol. **183** , 7341–7353. (10.1128/JB.183.24.7341-7353.2001) 11717293 PMC95583

[50] Schulthess B , Bloes DA , Berger-Bächi B . 2012 Opposing roles of σB and σB-controlled SpoVG in the global regulation of esxA in Staphylococcus aureus. BMC Microbiol. **12** , 17. (10.1186/1471-2180-12-17) 22272815 PMC3313859

[51] Casabona MG , Buchanan G , Zoltner M , Harkins CP , Holden MTG , Palmer T . 2017 Functional analysis of the EsaB component of the Staphylococcus aureus type VII secretion system. Microbiology **163** , 1851–1863. (10.1099/mic.0.000580) 29165232 PMC5845737

[52] Alcock F , Palmer T , Yang Y , Scott A . 2024 NanoBit assay data [dataset]. Figshare. (10.6084/m9.figshare.25368598)

[53] Yang Y , Scott AA , Kneuper H , Alcock F , Palmer T . 2024 Data from: High throughput functional analysis provides novel insight into type VII secretion in Staphylococcus aureus. Figshare. (10.6084/m9.figshare.c.7397761) PMC11322744 39139050

